# Soil Microbial Legacy Overrides the Responses of a Dominant Grass and Nitrogen-Cycling Functional Microbes in Grassland Soil to Nitrogen Addition

**DOI:** 10.3390/plants11101305

**Published:** 2022-05-13

**Authors:** Minghui Zhang, Xueli Li, Fu Xing, Zhuo Li, Xiaowei Liu, Yanan Li

**Affiliations:** 1 Key Laboratory of Vegetation Ecology, Ministry of Education, Jilin Songnen Grassland Ecosystem National Observation and Research Station, Institute of Grassland Science, Northeast Normal University, Changchun 130024, China; zhangmh740@nenu.edu.cn (M.Z.); lixl413@nenu.edu.cn (X.L.); liz347@nenu.edu.cn (Z.L.); liuxw101@nenu.edu.cn (X.L.); liyn100@nenu.edu.cn (Y.L.); 2 Jilin Provincial Key Laboratory of Ecological Restoration and Ecosystem Management, Northeast Normal University, Changchun 130024, China

**Keywords:** *Leymus chinensis*, soil microbial legacy, grassland succession, nitrogen deposition, soil nitrogen-cycling functional microbes, soil properties

## Abstract

Both atmospheric nitrogen (N) deposition and soil microbial legacy (SML) can affect plant performance, the activity of soil N-cycling functional microbes and the relative abundance of N-cycling functional genes (NCFGs). In the grassland vegetation successional process, how the interaction of SML and N deposition affects the performance of dominant grass and NCFGs remains unclear. Therefore, we planted *Leymus chinensis*, a dominant grass in the Songnen grassland, in the soil taken from the early, medium, late, and stable successional stages. We subjected the plants to soil sterilization and N addition treatments and measured the plant traits and NCFG abundances (i.e., *nifH*, AOB *amoA*, *nirS*, and *nirK*). Our results showed the biomass and ramet number of *L. chinensis* in sterilized soil were significantly higher than those in non-sterilized soil, indicating that SML negatively affects the growth of *L. chinensis*. However, N addition increased the plant biomass and the AOB *amoA* gene abundance only in sterilized soils, implying that SML overrode the N addition effects because SML buffered the effects of increasing soil N availability on NCFGs. Therefore, we emphasize the potential role of SML in assessing the effects of N deposition on dominant plant performance and NCFGs in the grassland vegetation succession.

## 1. Introduction

There is increasing interest in the importance of soil microbial legacies and their role in ecological succession [1,2]. Generally, previous plant communities have a long-lasting effect on soil microorganisms through the root exudate and litter, thus forming specific microbial communities [3]. Furthermore, these specific microbial communities critically impact the growth of subsequent plants, and this microbe-mediated effect is called the soil microbial legacy [3,4]. In many ecosystems, soil microbial legacy can influence the biomass of plants, regulate the contents of plant carbon (C), nitrogen (N) and phosphorus (P), and influence the replacement of plant species over time [1,4]. Many studies have concluded that soil microbial legacy affects plant growth through plant-microbe interactions [1,5] and potentially drives ecological succession through soil pathogen accumulation [6], soil nutrition changes [7], and the effects of symbiotic microbes [5]. Additionally, some studies have confirmed that soil microbial legacy regulates the changes in plant communities via soil microorganisms [3,8], whereas how the soil microbial legacy of ecological succession affects dominant species is still poorly understood. Therefore, it is worthwhile to elucidate the mechanisms of soil microbial legacy effects on ecosystem succession.

Soil microbial legacies play a key role in grassland vegetation successions because grassland ecosystems are highly sensitive to changes in the soil microenvironment [9]. In the early successional stages of grassland ecosystems, pioneer species produce soil microbial legacy effects by influencing soil microbes. These effects potentially impact the invasion, colonization, and growth of subsequent plants inhabiting the same area [10]. After mid-successional stages (i.e., plant species abundance and total biomass are significantly higher than those in early successional stages), soil microbial legacy often causes soil pathogen accumulation, thus decreasing the biomass of dominant species and promoting community turnover [6]. However, some studies report that symbiotic microorganisms will alleviate the negative effects of soil microbial legacy on some dominant species in the late successional stage [5,11]. Likewise, different plant species have diverse responses to the same soil microbial legacy due to the complexity of soil microbial community composition and structure in grasslands [12]. Therefore, it remains unclear whether the soil microbial legacy has positive or negative effects on dominant species during the successional stages of grassland ecosystems.

Soil N-cycling functional microbes are the main factors by which soil microbial legacy affects plant growth [13,14]. Due to the positive correlation between soil N-cycling functional microbes and the abundances of N-cycling functional genes (NCFGs), the expression of NCFGs can reflect how soil microbial legacy affects plant growth through the N-cycling functional microbes [13,15,16]. For instance, some plants can modify the composition of soil ammonia-oxidizing bacterial genes (AOB *amoA*) and alter the N cycle, thus forming the soil microbial legacy that influences subsequent plant growth [13]. Likewise, the expression of soil denitrifying microbial genes (*nirK* and *nirS*) indicates that crops respond to previous crop rotations [16]. Although previous plant communities are the main factor that creates soil microbial legacy, the effect of soil microbial legacy in grassland ecosystems is also influenced by atmospheric N deposition [17,18]. In particular, N deposition directly affects the soil N cycle and indirectly affects the N cycle-mediated soil microbial legacy in grasslands [18].

N deposition can impact the soil N cycle and grassland vegetation succession by increasing soil N availability, changing the proportion of soil C, N and P, and decreasing soil pH [18,19]. Some studies have emphasized that N deposition is a necessary condition for the formation of soil microbial legacies [17,20]. For instance, soil legacy effects created by long-term N deposition will impact the vegetation composition [21]. Therefore, how soil microbial legacy interacts with subsequent N deposition is a critical question to be resolved for predicting future plant responses to a changing environment [22]. Noteworthily, some evidence indirectly showed interactions between the N deposition and the soil legacy [23,24]. For example, N addition increased perennial rhizomatous grasses in a heavily grazed area but increased annuals in an ungrazed area [24]. This finding may be due to the effects of N deposition on plant–soil interactions being dependent on the previous soil environment [25,26]. However, few studies have examined the interaction between soil microbial legacy and N deposition in successional grasslands, and the mechanism of these interactions thus remains unclear.

The Songnen grassland is located in the northeastern plains of China. More than 40% of these grasslands are dominated by *Leymus chinensis* [27]. *L. chinensis* is a perennial rhizomatous grass with strong tolerance to drought, high pH, and low fertility [28]. As a pioneer species of Songnen grassland restoration succession, the growth of *L. chinensis* may be affected by both soil microbial legacy and atmospheric N deposition. This study examined the effects of N cycle-mediated soil microbial legacy on *L. chinensis* and the interaction between soil microbial legacy and N deposition on soil N cycle in a grassland vegetation successional series. We conducted a mesocosm experiment using *L. chinensis* and the soils of four different successional stages, and N addition was used to simulate N deposition. Some evidence has indicated that most soil legacy negatively affects subsequent plants [4,29]; likewise, NCFGs can reflect the N cycle-mediated soil microbial legacies [13,16]; moreover, soil legacy has a buffering capacity for soil environmental change [30]. Therefore we hypothesized that (1) the N cycle-mediated soil microbial legacies negatively affect *L. chinensis* growth by regulating NCFG abundances; (2) the soil microbial legacy overrides the effects of N addition on NCFG abundances and plant growth through the interaction between plants and NCFGs instead of soil properties. This study provides an insight into how the soil microbial legacy affects the growth of dominant species under the atmospheric N deposition.

## 2. Results

### 2.1. The Effects of Successional Stages and Sterilization on L. chinensis and NCFGs

**Figure 1:**
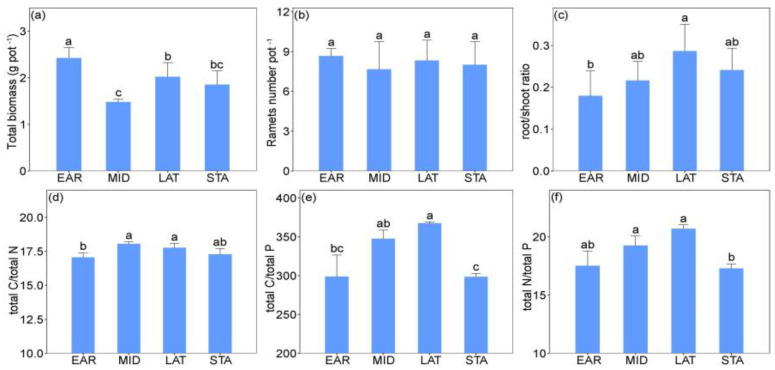
The differences in the traits of *L. chinensis* among the four successional stages. (**a**) plant total biomass; (**b**) ramets number; (**c**) root/shoot ratio; (**d**) total C/total N; (**e**) total C/total P; (**f**) total N/ total P. According to the Tukey’s test, different letters indicate significance among the four successional stages (*p* < 0.05). Vertical bars represent mean ± SE, *n* = 5. EAR, early successional stage; MID, middle successional stage; LAT, late successional stage; STA, stable community stage. total C, total carbon; total N, total nitrogen; total P, total phosphorus; total C/total N, total C/total P, and total N/total P indicate their ratios. Different letters indicate significant (*p* < 0.05) among different successional stages.

The total biomass, root/shoot ratio, total C/total N, total C/total P, and total N/total P of *L. chinensis* were significantly different across the different successional stages (SU) (Table 1). The total biomass in the early successional stage (EAR) was significantly higher than that in the middle successional stage (MID), late successional stage (LAT) and stable successional stage (STA) (Figure 1a). The total C/total N in the MID and LAT stages was significantly higher than that in the EAR stage (Figure 1d). Finally, total C/total P and total N/total P in the MID and LAT stages were significantly higher than those in the STA stage (Figure 1e,f).

**Figure 2:**
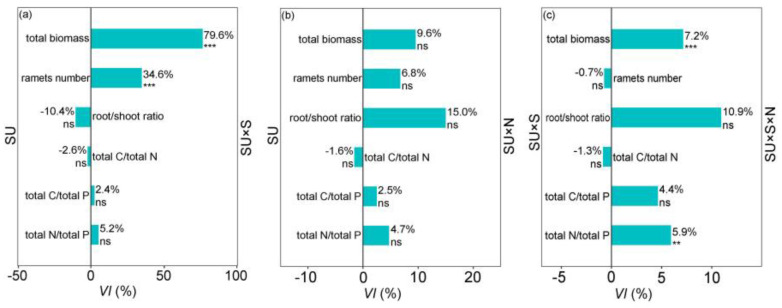
Variability index (*VI*) of plant traits in different treatment combinations of successional stages (SU), sterilization (S), and N addition (N). SU vs. SU × S (**a**) indicate the changes of plant traits after we eliminate soil microbial legacy effects; SU vs. SU × N (**b**) indicate the impact of N addition on plant traits (with soil microbial legacy effects); SU × S vs. SU × S × N (**c**) indicate the N addition effect on plant traits (without soil microbial legacy effects). The percentages indicate the *VI* of different traits of *L chinensis*; ***, *p* < 0.001; **, *p* < 0.01; *, *p* < 0.05; ns, no significant difference.

Soil sterilization (S) significantly affected the *L. chinensis* traits. Specifically, the interaction between SU and S significantly affected the total biomass, ramets number, total C/total N, total C/total P, and total N/total P (Table 1). In the SU × S treatment, the plant total biomass and ramets number were significantly different from those in the SU treatment (Figure 2a). The variability index (*VI*) values showed that the plant total biomass and ramets number in sterilized soils were 79.6% and 34.6% higher than those in non-sterilized soils (Figure 2a).

**Figure 3:**
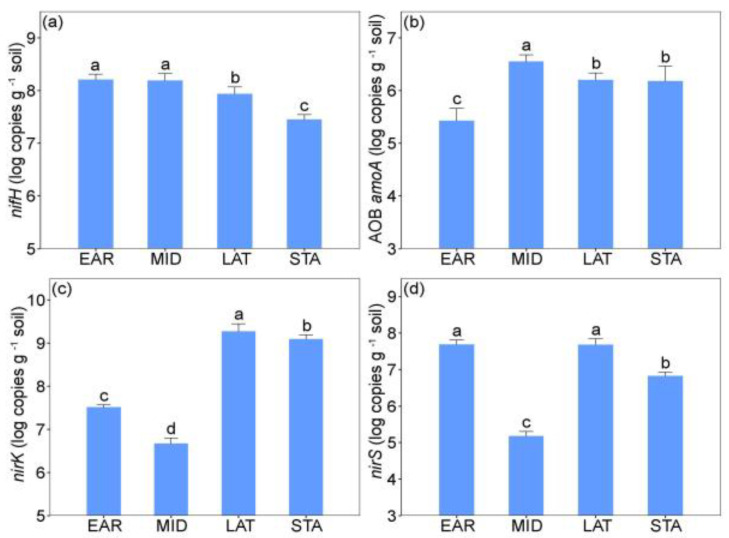
The differences of N-cycling functional gene abundances among the four successional stages. (**a**) *nifH* gene abundance; (**b**) AOB *amoA* gene abundance; (**c**) *nirK* gene abundance; (**d**) *nirS* gene abundance. According to the Tukey’s test, different letters indicate significant among the four successional stages (*p* < 0.05). Vertical bars represent mean ± SE, *n* = 5. EAR, early successional stage; MID, middle successional stage; LAT, late successional stage; STA, stable community stage. Different letters indicate significant (*p* < 0.05) among different successional stages.

The abundances of all four NCFGs were significantly different among the four successional stages (Table 2). Compared to the LAT and STA stages, significantly higher values of *nifH* gene abundances were found in the EAR and MID stages (Figure 3a). The abundances of AOB *amoA* gene in the EAR stage were significantly lower than those in other successional stages (Figure 3b). The *nirK* gene abundances in the LAT and STA stages were significantly higher than those in the EAR and MID stages (Figure 3c). Likewise, the *nirS* gene abundances in the EAR and LAT stages were significantly higher than those in the MID and STA stages (Figure 3d).

**Figure 4:**
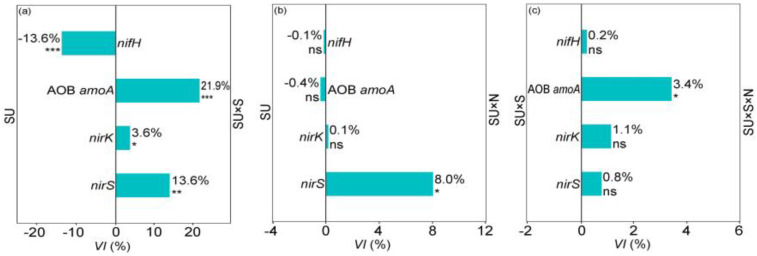
Variability index (*VI*) of N-cycling functional gene abundances (NCFGs) on the different treatments. SU, Successional stages; S, Sterilization; N, N addition. SU vs. SU × S (**a**) indicate the changes of NCFGs after we eliminate soil microbial legacy effects; SU vs. SU × N (**b**) indicate the impact of N addition on NCFGs (with soil microbial legacy effects); SU × S vs. SU × S × N (**c**) indicate the N addition effect on NCFGs (without soil microbial legacy effects). The percentages indicate the *VI* of different plant traits, significant difference are shown in: ***, *p* < 0.001; **, *p* < 0.01; *, *p* < 0.05; ns, no significant difference.

The interaction between SU and S significantly affected the abundances of all four NCFGs (Table 2). The *VI* value showed that, compared with NCFG abundances in non-sterilized soils, the *nifH* gene abundances significantly decreased by 13.6% after soil sterilization. However, the AOB *amoA*, *nirK*, and *nirS* gene abundances in sterilized soils were 21.9%, 3.6%, and 13.6% higher than in non-sterilized soils, respectively (Figure 4a).

### 2.2. Soil Microbial Legacy Altered by N Addition

The interaction between SU and N significantly affected plant root/shoot ratio, while SU, S, and N had significant interaction effects on the plant ramets number, root/shoot ratio, total C/total P, and total N/total P (Table 1). There was no significant difference in *L. chinensis* traits between SU treatment and SU × N treatment (Figure 2b). However, the plant total biomass and total N/total P in the SU × S × N treatment were significantly higher than that in the SU × S treatment. In sterilized soils, N addition significantly increased plant total biomass and total N/total P by 7.2% and 5.9%, respectively (Figure 2c).

The interaction between SU and N significantly affected the *nifH*, AOB *amoA*, and *nirS* gene abundance. SU, S, and N had a significant interaction effect on *nirK* and *nirS* gene abundance (Table 2). The *VI* value showed that the *nirS* gene abundance in the SU × N treatment was significantly increased by 8.0% when compared those in the SU treatment (Figure 4b). Moreover, the AOB *amoA* gene abundance in the SU × S × N treatment was significantly higher than that in the SU × S treatment. Finally, N addition significantly increased the AOB *amoA* gene abundance by 3.4% in sterilized soils (Figure 4c).

### 2.3. Key Factors and Pathways Influencing L. chinensis

**Figure 5:**
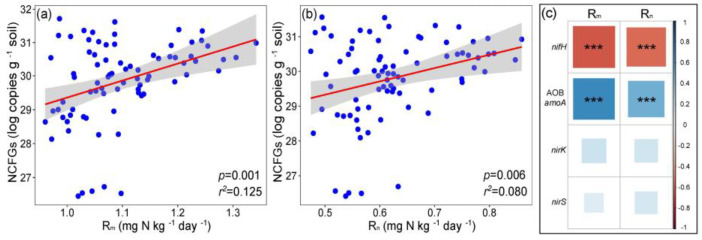
Net mineralization rate (Rm) and net N nitrification rate (Rn) in relation to N-cycling functional gene abundances (NCFGs). (**a**), the total relationship between NCFGs and Rm; (**b**), the total relationship between NCFGs and Rn; (**c**), the details of each N-cycling functional gene in relation to Rm and Rn; ***, *p* < 0.001.

**Figure 6:**
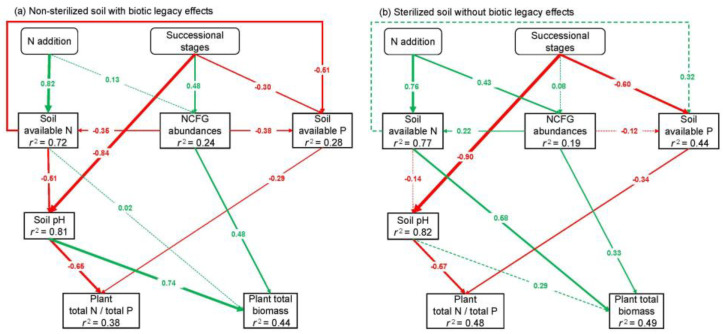
Structural equation models revealed the effects of successional stages and N addition on soil available nitrogen (N) and phosphorus (P), soil pH, N-cycling functional gene (NCFG) abundances, plant total biomass and total N/total P. Green and red arrows indicate positive and negative relationships, respectively. Continuous and dashed arrows indicate significant (*p* < 0.05) relationships and no significant (*p* > 0.05) relationships, respectively. Numbers on arrows are standardized path coefficients. The thickness of the arrow indicates the strength of the relationship. *r*^2^ values associated with response variables indicate the proportion of variation explained by relationships with other variables. Results of model fitting: (**a**) chisq = 0.238, rmsea = 0.000, srmr = 0.008, cfi = 1.000, *p* value = 0.626, (**b**) chisq = 1.060, rmsea = 0.050, srmr = 0.014, cfi = 0.999, *p* value = 0.303.

Pearson correlation showed that the total abundances of NCFGs positively correlated with the net N nitrification rate (Rn) and the net mineralization rate (Rm) (Figure 5a,b). Furthermore, *nifH* gene abundance positively correlated with Rn and Rm, but AOB *amoA* gene abundance negatively correlated with Rn and Rm (Figure 5c). The structural equation models (SEM) showed that the successional stages (SU) and N addition were significantly correlated with plant total biomass and plant total N/total P (Figure 6). In non-sterilized soils, SU negatively affected soil available P and pH, and thus influenced plant total N/total P. Moreover, SU positively affected NCFG abundance, and the NCFG abundances positively affected the plant total biomass (Figure 6a). After soil sterilization, SU still exerted negative effects on plant total N/total P via soil available P and pH. However, in contrast to the non-sterilized soil, soil pH was not significantly correlated with plant total biomass. Additionally, the NCFG abundances retained their positive effect on plant total biomass in sterilized soils, but there was no significant correlation between SU and the NCFG abundances (Figure 6b). In non-sterilized soils, N addition positively affected soil available N. The increase in soil available N was negatively correlated with soil pH and available P, and thus indirectly influenced plant total biomass and total N/total P (Figure 6a). After soil sterilization, N addition positively affects plant total biomass through increasing soil available N. However, soil pH and available P were not correlated with soil available N in the sterilized soil. Moreover, N addition influenced plant total biomass through its positive correlation with the NCFG abundances in sterilized soils (Figure 6b).

## 3. Discussion

### 3.1. The Effects of Soil Microbial Legacy on L. chinensis and NCFGs

Soil microbial legacy is created by previous plant communities and critically influences subsequent plant growth through soil microbes [1,5]. In this study, soil microbial legacy during grassland vegetation succession inhibited the growth of *L. chinensis* (Figure 1a and Figure 2a). This result is consistent with a previous study, which showed that soil microbial legacy decreased plant biomass [5]. The negative effect of soil microbial legacy promotes vegetation succession in the early successional stage and thus enhances the plant species richness of the grassland [3,31]. However, the soil microbial legacy also negatively affects future individuals of the same species and is not conducive to a single plant species dominating in the late successional stage [32]. Therefore, the negative effects of soil microbial legacy on dominant *L. chinensis* may inhibit the formation of stable *L. chinensis* communities.

We found that the NCFGs are critical factors of soil microbial legacy affecting plant growth (Figure 6a), similarly to a recent study in which land-use legacy effects were significantly correlated with NCFG abundances [33]. Previous plant species and biomass may regulate the composition of soil microbial communities [3], and the soil microbial legacy caused by them will further impact the interaction between NCFGs and subsequent plants [16,34]. In detail, after the soil microbial legacy was eliminated, the ammonia-oxidizing bacterial gene (AOB *amoA*) and denitrifying microbe gene (*nirS* and *nirK*) increased significantly (Figure 4a). This result implies that soil microbial legacy can limit the processes of soil ammonia oxidation and denitrification [35]. Moreover, some studies have demonstrated that the expression of AOB *amoA*, *nirK*, and *nirS* are correlated with the rates of soil N cycle and positively impact the microbe-mediated N absorption of the plant [16,36,37]. As a result, the low expression of AOB *amoA*, *nirK*, and *nirS* genes may be a reason why soil microbial legacy inhibited the growth of dominant *L. chinensis*.

### 3.2. Soil Microbial Legacy Overrides N Addition Effects on L. chinensis and NCFGs

We found that the *L. chinensis* traits and NCFG abundances (except the *nirS* gene) did not significantly respond to N additions in non-sterilized soils (Figure 2b and Figure 4b). Our result indicates that soil microbial legacy from different successional stages has a buffering capacity for N deposition. Some evidence demonstrated that low concentrations of N addition enhanced plant growth and NCFG abundances [38,39]. However, our finding was different from these studies. The reason may be that the effects of soil environmental changes on soil microbial communities and soil N cycle were overwhelmed by the soil microbial legacy [40]. Consequently, plant growth and NCFGs showed a lag response to soil environmental changes in non-sterilized soils [30]. Furthermore, our results showed that N deposition promotes the biomass of *L. chinensis* and AOB *amoA* gene abundance in sterilized soils (Figure 3c and Figure 4c). This result is similar to a finding in temperate grasslands [41]. Thus, plant growth benefits from nitrification to obtain soil N [35]. However, in the present study, AOB *amoA* had no response to N addition in non-sterilized soils (Figure 4b). Hence, we speculated that soil microbial legacy could maintain the stability of ammonia-oxidizing bacterial communities [35,42]. In addition, soil sterilization breaks the composition of soil microbial communities and eliminates the effects of soil microbial legacy [43]. Therefore, N addition directly affected plant growth and NCFGs (especially AOB *amoA* genes) in sterilized soils. Overall, we conclude that the soil microbial legacy overrides the effects of N addition on the performance of *L. chinensis* and the abundances of NCFGs by comparing the results of N addition in non-sterilized versus sterilized soils.

Furthermore, our findings showed that soil microbial legacy did not alleviate the effects of N addition on *nirS* gene abundance (Figure 4b). In detail, *nirS* gene abundance positively correlates with the process of soil denitrification, especially in grassland soil [37,44]. Some studies indicated that soil denitrification did not respond to previous plant species richness but did respond positively to changes in the soil abiotic factors (especially soil N) [45,46]. Therefore, although the soil N cycle is regulated by soil microbial legacy, N addition can still affect the denitrification process by increasing soil N nutrition and thus influencing the *nirS* gene abundance in non-sterilized soil.

### 3.3. Key Factors Affecting L. chinensis and NCFGs

Our results showed a significant correlation between NCFGs and the process of soil N mineralization (Figure 5). This finding is similar to a study in which the expression of NCFGs can reflect changes in the microbe-mediated soil N cycle [47]. However, we found that the abundance of the *nifH* gene is negatively associated with soil N mineralization. The reason may be that *nifH* gene abundance determines the nitrogen-fixing process of microorganisms, but the *nifH* gene is not involved in the microbial nitrification process [48]. As such, there is no evidence that the N mineralization rates will influence the capability of microbial N fixation. Additionally, some reports have shown that the N nutrition of plant acquisition depends on abiotic and microbial pathways [49,50]. The abiotic pathway represents the direct N absorption of plants in the soil and depends on the contents of soil N availability [51]. The microbial pathway reflected that plants indirectly acquire nutrients through N-cycling functional microbes, involving plants exporting C to soil microorganisms in exchange for N [25]. Likewise, the microbial pathway of plant N nutrient absorption can change soil N mineralization rates through plant-microbe interactions and therefore influence the expression of NCFGs [16,36]. Hence, the NCFG gene abundances and the relationships between NCFGs and plant performance provide indicators for assessing the microbial pathway of plant N nutrient acquisition.

The SEM results demonstrated that soil pH, available N, and available P were the main abiotic factors affecting plant performance (Figure 6). These results are consistent with some studies showing that soil nutrient availability and pH significantly affected plant performance [52,53,54]. Furthermore, the increasing soil available N caused by N addition indirectly affects plant performance through available P and pH. The reason may be that N addition caused soil acidification and aggravated P limitation and thus impacted plant growth [44,55]. Moreover, previous studies have shown that soil sterilization is the most effective way to eliminate the soil microbial legacy, but sterilization also releases soil N availability [3,43]. Sterilization may reduce the effects of N addition on plant growth because high N concentrations in soil environments make plant performance less susceptible to nitrogen augmentation [56]. However, our results showed that N addition directly affected plant growth through soil available N but only in sterilized soil. Therefore, sterilization did not interfere with our assessment of the relationship between the soil microbial legacy and N addition. Consequently, we propose that soil microbial legacy can alleviate the direct effect of N addition on plant performance, even if the non-sterilized soils contain low N availability.

Our results showed that soil microbial legacy across different successional stages affects the growth of *L. chinensis* via NCFGs, and N addition had no direct effect on NCFG abundances in non-sterilized soils (Figure 6). Our findings support the view that soil microbial communities critically mediate soil microbial legacy [8,57,58]. Generally, this soil microbial legacy is created by the microbe-mediated N absorption of previous plants, especially in grasslands where soil N is limiting [59,60]. In detail, previous plants influence plant-microbial interactions to absorb more N nutrients, maintaining the stabilization of previous microbial communities (especially soil N-cycling functional microbes) in a changing soil environment [33,61], thus forming the soil microbial legacy [62,63]. Finally, the soil microbial legacy potentially impacts dominant plant performance through N-cycling functional microbes [13,15] and buffers the effects of N addition on NCFGs.

In this study, we conducted a greenhouse experiment in one growing season to test the soil microbial legacy, and the effectiveness of this method has been confirmed in previous studies [8,64]. Additionally, a previous study showed the advantage of pot experiments for examining individual plant species in response to soil legacies [43]. Accordingly, it is reasonable to explore the effects of soil microbial legacy on unique plant species through pot experiments. Moreover, our study did not include the effects of allelopathy and interspecific competition in plants. Admittedly, plant roots can secrete allelochemicals (e.g., phenolic acids and flavonoids) into the soil [65]. If these metabolites reach sufficient concentration and retention time in the soil, they will affect the growth of subsequent plants [15,65]. In addition, allelochemicals exuded by plants can directly impact the composition of microbial communities, thus creating soil legacies and influencing subsequent plants [13,15]. Likewise, in the field area, the effects of soil microbial legacy on plant growth may be more complex due to interspecific competition. Therefore, further studies still need to be considered for the results of allelopathy and interspecific competition on soil legacy.

## 4. Materials and Methods

### 4.1. Sampling Area Description

The study was conducted at the Tongyu Semi-Arid Climate-Environment Field Station (122°52′ E, 44°25′ N), which is part of the Chinese Academy of Sciences, located in the Songnen Plain, northeastern China. The Tongyu station covers an area of 477,000 m^2^ and has been fenced since the autumn of 2006 to prevent grazing. The zonal vegetation type is described as meadow steppe, and the dominant plant species is *L. chinensis*. The mean annual precipitation in the region is 404 mm, with 80% occurring from May to September, which coincides with the growing season. The mean annual temperature is 5.7 °C, with the lowest of −32 °C in December and the highest of 38.9 °C in June. The topography is flat, and there is strong soil heterogeneity that includes sandy soils, slight chernozem soils, salty alkaline soils, and meadow soils [29].

Before fencing was established at Tongyu station, this area had different grazing intensities (i.e., heavy grazing centered around the water sources). As a result, grasslands in this area vary in terms of their degradation status [27]. Likewise, recovery was heterogeneous and depended on soil type and the vegetation degradation status [31]. Thus, this recovery formed a “successional series” that contains multiple successional stages [66]. We selected four different successional stages in this “successional series”, including the early successional stage (EAR), the middle successional stage (MID), the late successional stage (LAT), and the stable community stage (STA), at Tongyu station. Different successional stages were identified by the indicators for grassland degradation [67,68] and the guidelines for rangeland degradation assessment [69]. The presence of these successional stages was confirmed by our field investigation in August 2019. The plant communities corresponding to the four successional stages were named the *Chloris virgate* + *Kochia sieversiana* community, *L. chinensis* + forbs community, *L. chinensis* + *Lespedeza daurica*, and *L. chinensis* community (Table 3) [70]. The EAR of *C. virgate* + *K. sieversiana* community had low plant diversity and mainly consisted of saline-alkali tolerant plants. In the MID community, the plant species were obviously more abundant, and *L. chinensis* had invaded and became the dominant species. However, the biomass of forbs was greater than the biomass of *L. chinensis* in the MID community. In the LAT community, the dominant species switched to *L. chinensis* and *L. daurica*, and the total biomass of *L. chinensis* was significantly enhanced compared with that in the EAR and MID. The STA community had a few plant species, and the dominant species switched to *L. chinensis*. With grassland restoration, the soil pH gradually decreased and the soil total C, total N, and total P gradually increased (Table 4).

### 4.2. Experimental Soil Preparation and Vegetation Survey

We selected the four successional stages (EAR, MID, LAT and STA) at Tongyu station. Four identical plant communities spaced at least 200 m apart were selected as the four blocks for each successional stage. Within the 50 m × 50 m region of each block, we randomly selected four 1 m × 1 m sampling sites. After removing the litter on the soil surface, 0.5 kg of soil was collected from 0–0.2 m depth at each sampling site using a soil auger (0.04 m diameter). Finally, 2 kg of soil was randomly collected from the four sampling sites and mixed together as a soil sample for each block. Consequently, we obtained 8 kg of soil from each successional stage. Because the upper 0–0.2 m of the soil encompasses most of the above plant community’s roots, the collected soils were sufficiently conditioned by the plant community [58]. These soils were then transported to the laboratory and sieved (2 mm) to eliminate soil invertebrates, and half of the sample was separated and sterilized with an autoclave (121 °C, 0.5 MPa, 60 min). Sterilized and unsterilized soils were used for the cultivation of *L. chinensis*.

On 15 August 2019, a field vegetation survey was conducted at each successional stage. One 1 m × 1 m plot was established near the above sampling sites. Therefore, there were four repeated vegetation survey plots at each successional stage. All plants in each plot were harvested, and then the average density, average height, aboveground biomass, and coverage of each plant species were measured. Aboveground biomass was measured by clipping all plants, drying at 60 °C for 48 h, and weighing the dried samples. Following the vegetation surveys, soil samples were collected using a soil auger (0.04 m diameter) from the upper 0–0.2 m of soil at three randomly selected points in each plot and combined to form a composite sample for analyzing soil properties.

### 4.3. Mesocosm Experimental Design and Execution

We used a randomized block design with three factors, including successional stage (i.e., EAR, MID, LAT, and STA), N addition (i.e., N not added and N added), and soil sterilization (i.e., sterilized soil and non-sterilized soil). We planted *L. chinensis* in soils collected from the four different successional stages. The N fertilizer that we used was NH_4_NO_3_, which had an N content of 35%. Then, 0.144 g of NH_4_NO_3_ was added to each pot. We sterilized the soils to eliminate soil microbial legacy effects. In addition, we used an N addition treatment to explore how N addition affects soil microbial legacy effects. Overall, we had a total of 16 (4 × 2 × 2) treatment combinations and five replicates for each treatment (Figure S1).

On 15 August 2018, *L. chinensis* seeds were collected from the *L. chinensis* grassland community at Tongyu station. On 1 April 2019, the *L. chinensis* seeds were surface-sterilized for 1 min using 1% sodium hypochlorite, rinsed, and sown in the nursery with sterilized soil, and seedlings were grown in the greenhouse at Jilin Songnen Grassland Ecosystem National Observation and Research Station, China (44°45′ N, 123°45′ E) (i.e., our greenhouse and laboratory mainly located here). On 1 May 2019, the four-week-old seedlings were transplanted into pots (polyethylene material, 0.12 m diameter, and 0.14 m height). Before the seedlings were transplanted, each pot was filled with 1.5 kg of natural or sterilized soil collected from the different successional stages in the grassland ecosystem. Three *L. chinensis* seedlings were planted in each pot. N additions totaling 10 g·N·m^−2^·year^−1^ were then divided into three equal parts, dissolved in 100 mL of water, and added to the pots at the beginning of May, June, or July. Similarly, 100 mL of water was added to each control pot. All the pots were randomly placed in a greenhouse (28 °C ± 3 °C daytime temperature, 19 °C ± 3 °C at nighttime temperature, relative humidity of 45% ± 5%, and maximum light intensity of 1900 μmol·m^−^^2^·s^−^^1^) and were turned once every two weeks. Distilled water was added to each pot once a week to ensure plant growth.

### 4.4. Sampling and Measurements of the Plants and Soil

After 12 weeks, plant and soil samples were collected on 1 August 2019. All *L. chinensis* was carefully removed from each pot, and the ramets number of each *L. chinensis* was recorded. The above- and belowground parts were separated and dried at 105 °C for 15 min and then at 65 °C for 48 h to measure the biomass. The total biomass and the root/shoot ratio of *L. chinensis* were then calculated using all *L. chinensis* in each pot. We ground the aboveground samples to produce particles size less than 1 mm. Next, total C was measured using a total organic carbon analyzer (Vario TOC, Elementar, Langenselbold, Germany), total N was determined using a Kjeldahl apparatus (Kjeltec 8400, FOSS, Hilleroed, Denmark), and total P was measured using an automatic discontinuous chemical analyzer (Smartchem 450, AMS, Guidonia, Italy). We also calculated total C/total N, total C/total P, and total N/total P using these data. There were five replicates for all plant traits.

Rhizosphere soils were collected using the “shaken off” method and sieved to 2 mm [71]. Then, soils from the same pot were mixed together to ensure the homogeneity of the soil sample in each pot. Afterward, each soil sample was divided into two equal parts. One part of the rhizosphere soil was air-dried to test the soil properties (i.e., pH, moisture, and the C, N and P contents). Soil pH was measured using a water suspension (water/soil = 5/1) and a pH meter (pH S-3C, INESA, Shanghai, China). Soil moisture was measured by drying the samples at 105 °C until a constant weight was reached. The methods used to measure the soil total C, soil total N, and soil total P were the same as those used for the plant samples. Soil available N was measured using a flow analyzer (Futura, AMS, Villeneuve-la-Garenne, France). Soil available P was determined using a spectrophotometer (UV5-500, METASH, Shanghai, China).

The second part of the rhizosphere soil sample was stored at −80 °C for the analysis of NCFG abundances and N mineralization rate. These NCFG genes included nitrogen-fixing bacterial genes (*nifH*), bacterial ammonia-oxidizing genes (AOB *amoA*), and bacterial denitrifying genes (*nirS* and *nirK*). Soil DNA was extracted using a PowerSoil^®^ Kit (DNeasy^®^ Powerlyzer^®^, Inc., Germantown, Tennessee, USA), and following the manufacturer’s protocol. DNA concentrations were measured using a NanoDrop 2000 to confirm the concentration of DNA. Samples were diluted to 20 ng DNA μL^−1^ using ultrapure water for quantitative real-time PCR (qPCR) measurements. Amplification of the qPCR products for NCFGs was assessed using a StepOne^TM^ Real-Time PCR System (Applied Biosystems, CA, Foster City, CA, USA). With the 10 μL Fast qPCR Master Mix (High Rox) (BBI, Beijing, China), 0.4 uL (200 nm) primers, and 20 ng template DNA were added, and the reaction mixture was made up to a final volume of 20 μL. The details of the qPCR reactions of the *nifH*, AOB *amoA*, *nirS*, and *nirK* genes are shown in Table S1.

The net N nitrification rate (Rn) and net mineralization rate (Rm) were estimated by aerobic incubation [72]. 10 g soil was placed in a glass flask, and then a perforated parafilm was covered on the top. The soil was incubated in the dark at 25 °C. After 20 days, the NH_4_^+^-N and NO_3_^−^-N concentrations of the incubated and non-incubated soils were measured using a flow analyzer (Futura, AMS, Villeneuve-la-Garenne, France). The Rn and Rm were derived from the following formulas:

$$\mathrm{Rn}=\frac{\mathrm{Incubated}\;({\mathrm{NO}}_{3})-\mathrm{Nonincubated}\;({\mathrm{NO}}_{3})}{20}\;\mathrm{Rm}=\frac{\mathrm{Incubated}\;({\mathrm{NH}}_{4}+{\mathrm{NO}}_{3})-\mathrm{Nonincubated}\;({\mathrm{NH}}_{4}+{\mathrm{NO}}_{3})}{20}$$

In these equations, incubated (NO_3_) and incubated (NH_4_ + NO_3_) represent the NO_3_^−^-N and the sum concentrations of NH_4_^+^-N and NO_3_^−^-N in the incubated soil, respectively. Nonincubated (NO_3_) and nonincubated (NH_4_ + NO_3_) represent the NO_3_^−^-N and the sum concentrations of NH_4_^+^-N and NO_3_^−^-N before the soil incubation, respectively. The number 20 represents the number of incubation days. The measurements of each soil properties, NCFGs, Rn, and Rm had five replicates.

### 4.5. Statistical Analyses

Three-way ANOVA was used to test for the effects of different successional stages (SU), sterilization (S), and N addition (N) on plant traits, NCFG abundances, and soil properties. The soil microbial legacy effects were assessed by comparing SU and SU × S (i.e., soil sterilization to eliminate soil microbial legacy effects). The impact of N addition on the soil microbial legacy effects was assessed by comparing SU and SU × N. The N addition effect (without soil microbial legacy effects) on plant traits and NCFG abundances was determined by comparing SU × S and SU × S × N.

To quantify soil microbial legacy effects, N addition, and its effects on interactions with plant traits and NCFG abundances (i.e., SU compared with SU × S, SU compared with SU × N, and SU × S compared with SU × S × N), we used the following formula to calculate the variability index (*VI*) of plant traits and NCFG abundances:

$$VI=100\%\times \frac{E_{B}-E_{A}}{E_{A}}$$

In the above equation, *E_A_* represents the mean pretreatment value for both sterilization and N addition, and *E_B_* represents the average posttreatment value. In other words, *VI* > 0 or *VI* < 0 represents the percent increase or decrease, respectively, after the treatment.

To testify the relationships between NCFG abundances and soil N mineralization rate, we conducted the correlation (Pearson) analyses with the NCFG abundances against soil Rn and Rm. The NCFGs include the abundances of *nifH*, AOB *amoA*, *nirS*, and *nirK* genes, and the total abundances of all four genes.

We used the structural equation model (SEM) to determine the key factors that significantly influenced plant growth and to analyze the interaction between soil microbial legacy effects and N addition. We treated successional stage (SU) and N addition as exogenous variables. Endogenous variables included all measured *L. chinensis* traits, the total abundance of NCFGs (including the abundances of the *nifH*, AOB *amoA*, *nirS*, and *nirK* genes), and all measured soil properties. We then selected the most important predictors according to the Akaike information criterion (AIC) [73]. The following were used to evaluate the fit of our models: the goodness of fit of the SEM was evaluated using a chi-square test (0 < chi-sq < 2, *p* > 0.05) and a root mean squared error of approximation (rmsea < 0.05). The standardized root mean square residual (srmr < 0.05) and comparative fit index (0.97 < cfi < 1) were used for the best-fitting SEM for each factor [74]. Significant differences in all statistical analyses were assessed using a *t* test at an alpha level of 0.05. All statistical analyses were conducted using R 4.0.2 [75].

## 5. Conclusions

The interaction between soil microbial legacy and N addition provides new insight into the mechanism that explains how the soil microbial legacy affects dominant *L. chinensis* under atmospheric N deposition. Our study demonstrates that soil microbial legacy negatively affected dominant *L. chinensis*. The negative effect of different successional stages for dominant *L. chinensis* may delay the grassland restoration into stable communities. Moreover, soil microbial legacy buffered the effects of N addition on NCFGs and plant performance, therefore promoting the asynchrony responses of soil abiotic factors, N-cycling functional microbes, and dominant plant growth to atmospheric N deposition. The buffering capacity of the soil microbial legacy explained the view that the dominant grass across the grassland successions does not respond to atmospheric N deposition in the short term. Consequently, we propose that soil microbial legacy decreased the effects of atmospheric N deposition on dominant grass.

## Figures and Tables

**Table 1:** Three-way ANOVA of the effects of different treatments on *L. chinensis* traits.

Plant Traits	SU		S		N		SU × S		SU × N		S × N		SU × S × N	
	*F*	*p*	*F*	*p*	*F*	*p*	*F*	*p*	*F*	*p*	*F*	*p*	*F*	*p*
total biomass	3.902	0.018	360.040	<0.001	7.691	0.009	2.940	0.048	2.661	0.065	0.001	0.970	1.592	0.210
ramets number	0.801	0.502	23.043	<0.001	0.064	0.802	3.241	0.035	0.461	0.711	0.574	0.454	1.161	0.340
root/shoot	2.178	0.110	9.278	0.005	0.014	0.907	1.959	0.140	2.755	0.059	0.224	0.639	4.882	0.007
total C/total N	14.387	<0.001	3.128	0.086	0.452	0.506	3.232	0.035	2.951	0.047	0.535	0.470	1.404	0.260
total C/total P	26.907	<0.001	3.691	0.064	0.802	0.377	4.776	0.007	1.457	0.245	0.550	0.464	4.727	0.008
total N/total P	15.960	<0.001	15.303	<0.001	2.940	0.096	5.482	0.004	0.307	0.820	0.148	0.703	12.648	<0.001

Treatments: Successional stages (SU), Sterilization (S), N addition (N), and their interactions. total C, total carbon; total N, total nitrogen; total P, total phosphorus; total C/total N, total C/total P, and total N/total P indicate their ratios. The significant values are defined as *p* < 0.05.

**Table 2:** Three-way ANOVA of the effects of different treatments on N-cycling functional gene (NCFG) abundances.

NCFG Abundances	SU		S		N		SU × S		SU × N		S × N		SU × S × N	
	*F*	*p*	*F*	*p*	*F*	*p*	*F*	*p*	*F*	*p*	*F*	*p*	*F*	*p*
*nifH*	30.853	<0.001	463.562	<0.001	0.083	0.775	4.864	0.007	1.697	0.187	0.935	0.341	0.292	0.831
AOB *amoA*	30.525	<0.001	440.357	<0.001	3.752	0.062	12.933	<0.001	1.325	0.283	4.397	0.044	0.846	0.479
*nirK*	329.179	<0.001	20.557	<0.001	1.091	0.304	20.197	<0.001	0.183	0.907	1.245	0.273	1.802	0.167
*nirS*	48.667	<0.001	58.224	<0.001	11.266	0.002	30.589	<0.001	11.870	<0.001	7.535	0.010	12.241	<0.001

Treatments: Successional stages (Su), Sterilization (S), N addition (N), and their interactions. *nifH*, nitrogen fixing bacteria; AOB *amoA*, ammonia oxidizing bacteria; *nirK* and *nirS*, denitrifying bacteria. The significant values are defined as *p* < 0.05.

**Table 3:** The vegetation characteristics of different successional stages in the grassland restoration processing (mean ± SE, *n* = 4).

Successional Stages	Community Type	Main Plant Species	Aboveground Biomass (g/m^2^)	Density (ramet/m^2^)	Height (m)	Coverage (%)
EAR	*Chloris virgata* + *Kochia sieversiana*	*C. virgata*	18.72 ± 11.67	192.50 ± 140.44	0.17 ± 0.05	34.50 ± 24.31
		*K. sieversiana*	82.61 ± 12.09	528.00 ± 338.83	0.23 ± 0.07	63.75 ± 22.87
		*Artemisia scoparia*	15.21 ± 7.55	72.50 ± 37.38	0.26 ± 0.07	15.00 ± 8.29
MID	*Leymus chinensis* + forbs	*L. chinensis*	148.69 ± 15.41	285.00 ± 46.35	0.35 ± 0.09	55.00 ± 14.72
		*Calamagrostis macrolepis*	3.45 ± 2.78	4.25 ± 2.30	0.34 ± 0.10	8.00 ± 5.12
		*Lespedeza daurica*	7.99 ± 6.08	10.50 ± 8.70	0.20 ± 0.08	3.75 ± 1.99
		*K. sieversiana*	2.07 ± 1.01	1.50 ± 0.91	0.28 ± 0.10	1.38 ± 0.43
		*A. scoparia*	1.31 ± 0.45	4.50 ± 2.65	0.26 ± 0.10	1.50 ± 1.00
		*Allium mongolicum*	1.33 ± 0.53	2.50 ± 1.65	0.30 ± 0.06	1.38 ± 0.80
LAT	*L. chinensis* + *L. daurica*	*L. chinensis*	227.22 ± 36.47	384.00 ± 36.37	0.49 ± 0.09	48.75 ± 14.45
		*C. macrolepis*	33.94 ± 41.58	62.00 ± 75.15	0.56 ± 0.09	8.75 ± 14.36
		*L. daurica*	70.27 ± 20.43	76.50 ± 38.99	0.49 ± 0.13	31.25 ± 16.52
STA	*L. chinensis*	*L. chinensis*	234.64 ± 35.61	431.00 ± 164.90	0.47 ± 0.09	85.00 ± 8.16
		*Carex duriuscula*	24.89 ± 12.96	612.00 ± 272.82	0.17 ± 0.05	30.00 ± 7.07

Note: EAR, early successional stage; MID, middle successional stage; LAT, late successional stage; STA, stable community stage.

**Table 4:** The soil properties of different successional stages in the grassland restoration processing (mean ± SE, *n* = 4).

Successional Stages	EAR	MID	LAT	STA
Soil pH	9.71 ± 0.11	8.77 ± 0.43	8.13 ± 0.29	8.46 ± 0.13
Soil moisture (%)	10.04 ± 0.00	8.86 ± 0.01	8.58 ± 0.01	11.37 ± 0.01
Soil total C (mg/kg)	7182.50 ± 1065.00	117,25.00 ± 907.06	12,130.00 ± 2150.66	12,480.00 ± 2170.44
Soil total N (mg/kg)	175.95 ± 12.69	222.70 ± 30.59	278.75 ± 17.33	235.15 ± 15.29
Soil total P (mg/kg)	212.80 ± 22.99	251.75 ± 18.13	238.30 ± 20.77	270.30 ± 13.10
Soil available N (mg/kg)	14.29 ± 1.93	15.85 ± 2.59	13.60 ± 2.27	12.10 ± 0.56
Soil available P (mg/kg)	1.38 ± 0.23	2.32 ± 0.20	1.22 ± 0.18	1.50 ± 0.12

Note: EAR, early successional stage; MID, middle successional stage; LAT, late successional stage; STA, stable community stage; C, carbon; N, nitrogen; P, phosphorus.

## Data Availability

The data that supports the findings of this study are contained within the article and available from the corresponding author upon reasonable request.

## Supplementary Materials

The following supporting information can be downloaded at: https://www.mdpi.com/article/10.3390/plants11101305/s1, Figure S1: The experimental framework of *L. chinensis* growth and soil N-cycling functional microbes in response to soil legacy and N addition across different successional stages; Table S1: Primers and reaction details of qPCR. References [76,77,78,79] are cited in the supplementary materials.
