# Supplementary material for: Soil Microbial Legacy Overrides the Responses of a Dominant Grass and Nitrogen-Cycling Functional Microbes in Grassland Soil to Nitrogen Addition
Source: Plants (Basel). 2022 May 13;11(10):1305. doi: 10.3390/plants11101305 (PMC9145027; doi:10.3390/plants11101305)
Supplement: Supplementary file 1 [file plants-11-01305-s001.zip › plants-1678393-supplementary.pdf]

Table S1. Primers and reaction details of qPCR.

Genes	Target/Primers	Sequence(5'-3')	Thermal Conditions	Reference
nifH	PolF	TGCGAYCCSAARGCBGACTC	94°C / 45 s, 55°C / 45 s, 72°C / 45 s, 40 cycles	[76]
	PolR	ATSGCCATCATYTCRCCGGA		
AOB *amoA*	amoA-1f	GGGGTTTCTACTGGTGGT	94°C / 60 s, 58°C / 60 s, 72°C / 60 s, 40 cycles	[77]
	amoA-2r	CCCCTCKGSAAAGCCTTCTTC		
nirK	F1aCu	ATCATGGTSCTGCCGCG	95°C / 45 s, 55°C / 45 s, 72°C / 45 s, 40 cycles	[78]
	R3Cu	GCCTCGATCAGRTTGTGGTT		
nirS	cd3AF	GTSAACGTSAAGGARACSGG	95°C / 15 s, 63° C / 30 s, 72°C / 30 s, 80°C / 30s, 35 cycles	[79]
	R3cd	GASTTCGGRTGSGTCTTGA		

76. Vadakattu, G.; Zhang, B.; Penton, C.R.; Yu, J.; Tiedje, J.M. Diazotroph diversity and nitrogen fixation in summer active perennial grasses in a mediterranean region agricultural soil. *Front. Mol. Biosci.* **2019**, *6*, 115.
77. Nogueira, C.B.; Menéndez, E.; Helena, M.; Bahena, R.; Velázquez, E.; Scotti, M.R. The N-fixing legume periandra mediterranea constrains the invasion of an exotic grass (*Melinis minutiflora* P. Beauv) by altering soil N cycling. *Sci. Rep.* **2019**, *9*, 11033.
78. Cuer, C.A.; Rodrigues, R.D.A.R.; Balieiro, F.C.; Jesus, J.; Silva, E.P.; Alves, B.J.R. Short-term effect of *Eucalyptus* plantations on soil microbial communities and soil-atmosphere methane and nitrous oxide exchange. *Sci. Rep.* **2018**, *8*, 15133.
79. Zhang, Y.; Hao, X.; Garcia-Lemos, A.M.; Nunes, I.; Nicolaisen, M.H.; Nybroe, O. Different Effects of Soil Fertilization on Bacterial Community Composition in the *Penicillium canescens* Hyphosphere and in Bulk Soil. *Appl. Environ. Microbiol.* **2020**, *86*, 1–16.

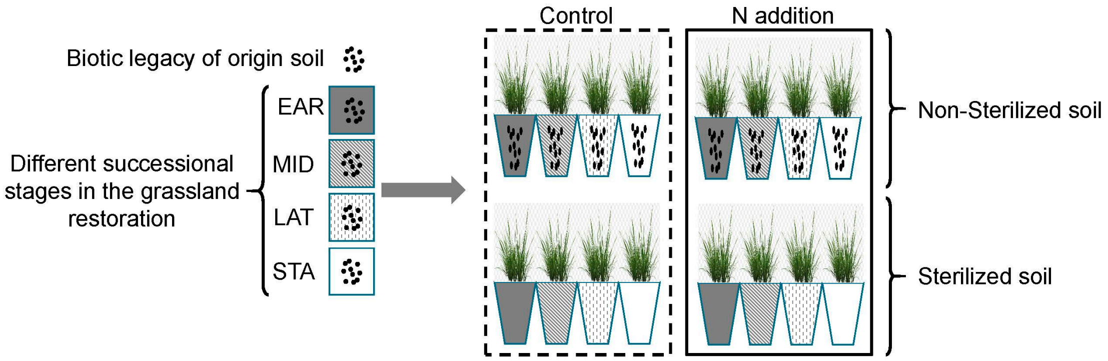

Figure S1. The experimental framework of *L. chinensis* growth and soil N-cycling functional microbes in response to soil legacy and N addition across different successional stages. EAR, early successional stage; MID, middle successional stage; LAT, late successional stage; STA, stable community stage. Dashed frame and continuous frame indicate without N addition (Control) and N addition. Black points indicate the biotic legacy of origin soil.
